# A Capsid Structure of *Ralstonia solanacearum podoviridae* GP4 with a Triangulation Number T = 9

**DOI:** 10.3390/v14112431

**Published:** 2022-11-01

**Authors:** Jing Zheng, Wenyuan Chen, Hao Xiao, Fan Yang, Xiaowu Li, Jingdong Song, Lingpeng Cheng, Hongrong Liu

**Affiliations:** 1 Institute of Interdisciplinary Studies, Key Laboratory for Matter Microstructure and Function of Hunan Province, Key Laboratory of Low-Dimensional Quantum Structures and Quantum Control, Hunan Normal University, Changsha 410082, China; 2 State Key Laboratory of Infectious Disease Prevention and Control, National Institute for Viral Disease Control and Prevention, Chinese Center for Disease Control and Prevention, Beijing 100052, China; 3 School of Electronics and Information Engineering, Hunan University of Science and Engineering, Yongzhou 425199, China

**Keywords:** *Ralstonia solanacearum* phage, icosahedral head, triangulation number T = 9, trimeric CP, Cryo-EM

## Abstract

GP4, a new *Ralstonia solanacearum* phage, is a short-tailed phage. Few structures of *Ralstonia solanacearum* phages have been resolved to near-atomic resolution until now. Here, we present a 3.7 Å resolution structure of the GP4 head by cryo-electron microscopy (cryo-EM). The GP4 head contains 540 copies of major capsid protein (MCP) gp2 and 540 copies of cement protein (CP) gp1 arranged in an icosahedral shell with a triangulation number T = 9. The structures of gp2 and gp1 show a canonical HK97-like fold and an Ig-like fold, respectively. The trimeric CPs stick on the surface of the head along the quasi-threefold axis of the icosahedron generating a sandwiched three-layer electrostatic complementary potential, thereby enhancing the head stability. The assembly pattern of the GP4 head provides a platform for the further exploration of the interaction between *Ralstonia solanacearum* and corresponding phages.

## 1. Introduction

The virion of tailed dsDNA phages consists of an isometric icosahedral or an elongated icosahedral head (or capsid) and a tail with a different morphology. The tail connects to the head via a portal complex occupying one of the 12 vertices of the icosahedral head. The phage head is necessary to accommodate the densely packed genome and protects the genome against harsh environments. The tail contains phage-receptor recognition proteins and penetrates the cell envelope and triggers DNA release from the head to the host shell [1,2,3]. The high symmetry of the head facilitates the acquisition of a high-resolution structure. In recent years, a series of icosahedral structures of phage capsids, herpesviruses and some dsDNA archaeal viruses were determined at near-atomic resolution by cryo-EM, leading to further insights into capsid construction and assembly [4,5,6,7,8,9]. The capsids mainly contain major capsid proteins (MCPs), whereas some phages and herpesviruses also encode additional small cement proteins (CPs) that interact with MCPs to enhance the capsid stability [10,11]. Despite possessing a mere 10% sequence identity, almost all known MCPs of tailed phages and the floor domain of herpesviruses show a similar structure, with a HK97-like fold that was first solved in the icosahedral head of HK97 by X-ray diffraction [12]. In most cases, the variation in phage heads involves only a difference in the copy numbers of MCP, which can be described by the triangulation (T) numbers [13,14]. Phage capsids with different T numbers, ranging from T = 3 in phi29 [15] to T = 52 in phage G [16], have been determined; most studies of capsid structure focused on T = 7, such as T7 [17], SF6 [18], ε15 [19], Pam1 [20], and BPP-1 [21].

The stabilization of the head is crucial for survival under adverse environments during the life cycle of a phage. The HK97 capsid is reinforced by isopeptide bonds between adjacent MCP subunits [12]. The capsids of P22, SF6, CUS-3, and A-1(L) are reinforced by sharing an extra MCP domain [18,22,23,24]. By contrast, the capsids of BPP-1 [21], Mic1 [25], ε15 [19], Lambda [26], P74-26 [27], and TW1 [28] are reinforced by encoding accessory gene products, called cement proteins (CPs) [10], binding to the capsid surfaces. Interestingly, the two typical categories of CPs include one located at the center of some capsomeres of capsids, such as the CPs of T5, Shigella [29], phiRSL1, Syn5 [30], and phiM5 [31], and the other located at the interface of capsomeres, such as CPs in BPP-1, Mic1 [25], ε15 [19], Lambda [26], P74-26 [27], and TW1 [28]. In particular, Phi812 [32], T4 [33,34], and phiM9 [35] not only carry CPs, but also have an extra domain in their MCPs, resulting in a unique connection pattern for capsid stabilization. In some cases, CPs are not involved in the assembly of the phage prohead but are added onto the outer surface of the phage capsid accompanied by genome packaging, resulting in significant functional enhancement [10,36,37,38].

Here, we isolated a new short-tailed double-stranded DNA phage, GP4, which infects *Ralstonia solanacearum*, from *Ralstonia* strains. Previous experimental results show that GP4 is a heat-resistant phage at temperatures ranging from 40 °C to 70 °C [39]. *Ralstonia solanacearum*, exhibiting an unusually wide host range, is a soil-borne Gram-negative bacterium, causing bacterial wilt in important field crops and resulting in a wide range of economical challenges worldwide. In the past decades, the majority of studies investigating the *Ralstonia solanacearum* phage focused on sequencing, genome analysis, and host range [40,41,42,43]. A few head structures of *Ralstonia solanacearum* phages, such as contractile tailed phages ϕRSA1 with T = 7 [44], ϕRSL2 with T = 27, ϕRP13 with T = 21 [45], and ϕRSL1 with T = 27 [46,47] have been reported; however, only the head of ϕRSA1 has been resolved to near-atomic resolution [44]. Moreover, the head information for *Ralstonia solanacearum* phages, which possess T value of 9, have not been reported. In the meantime, no high-resolution structure of short-tailed *Ralstonia solanacearum* phages is available, despite the completed genome sequence of short-tailed RPY1 [48], RsoP1EGY [49], and *Ralstonia* phage Cimandef [40] being reported. Hence, the structural information available to date regarding *Ralstonia solanacearum* phages limits our understanding of the complex relationship between *Ralstonia solanacearum* and the corresponding phages.

In this paper, we solved an icosahedral head structure of the bacteriophage GP4 by cryo-EM to 3.7 Å resolution, which enabled us to build the full atomic models of MCP gp2 and CP gp1. Our structure reveals that the icosahedral head of GP4 is composed of 540 copies of MCP gp2 and CP gp1 arranged in a shell with T value of 9. The specific structure of GP4 is perhaps the most intensive characterization of an *Ralstonia solanacearum* phage to date for the possible biocontrol of bacterial wilt disease.

## 2. Materials and Methods

### 2.1. Production and Purification of GP4

*Ralstonia* strain (Phagelux (Nanjing) Bio-tech Co., Ltd., Nanjing, China) was grown in TSB medium (15 g Tryptone, 5 g Soya Peptone, and 5 g sodium chloride per liter) for 48 h at 30 °C. *Ralstonia* GP4 phage was incubated with the cells for 15 min at 30 °C. The incubation mixture was inoculated with *Ralstonia* cells for 24 h at 30 °C. The cell lysate was removed by low-speed centrifugation at 6000× *g* for 20 min at 8 °C. The purified supernatant was enriched with 10% PEG8000 (Amresco, Solon, OH, USA) with 1 M NaCl (Aladdin, Shanghai, China) precipitation overnight at 4 °C. The precipitated particles were resuspended in SM buffer (10 mM Tris–HCl, pH 7.5, 100 mM NaCl, and 10 mM MgSO4) (Amresco, Solon, OH, USA). The resuspended particles were clarified by low-speed centrifugation and digested with DNase I and RNase (Beyotime, Shanghai, China). The phages were purified via two cesium chloride (CsCl) (Sigma, St. Louis, MO, USA) gradient centrifugation steps. The first step gradient phage was layered over CsCl gradient (1.45 g/mL CsCl, 1.50 g/mL CsCl, 1.70 g/mL CsCl) at 87,000× *g* for 2 h at 4 °C. Then, the mature phage of the lower band was collected for buoyant density gradient centrifugations. Then, the second continuous CsCl density gradient (1.50 g/mL CsCl) was performed at 150,000× *g* for 24 h at 4 °C. The purified bands were dialyzed against SM buffer overnight. After desalination, the final purified phages were stored in ice-water for cryo-sampling.

### 2.2. Cryo-EM Imaging and Image Processing

An aliquot of 3.5 µL purified GP4 was applied to a Quantifoil R2/1 copper grid, which had been glow discharged for 30 s. Grids were loaded in an FEI Vitrobot with several settings at 8 °C with 100% humidity, blotted for 2.5–4.0 s, and plunged into liquid ethane. The grid was stored in liquid nitrogen until data collection. The data were recorded using an FEI Tecnai Arctica 200-kV electron microscope equipped with a Falcon II camera at a normal magnification of 78,000×, which resulted in a pixel size of 1.27 Å. The accumulated dose of each movie was approximately 35 e^−^/Å^2^. Finally, a total of 3921 movies were collected and each movie stack consisted of 30 image frames [50]. The astigmatism and defocus value of each image were determined using GCTF wrapped in RELION-3.1.4 (Cambridge CB2 0QH, UK) [51]. The viral particles were boxed automatically using the software ETHAN [52], and then verified manually. A total of 40,792 particle images were extracted from the raw images and used to conduct icosahedral reconstruction. The determination of orientations and centers for all the particle images and 3D reconstructions were performed using our programs [53,54] based on the common-line algorithm [55,56]. For each particle image, the center parameter was estimated using self-correlation and next used to determine the angular parameter through a global search of the icosahedral asymmetric unit. This was performed based on minimizing the average amplitude-weighted phase residual (PR) of the common lines between the particle image and template projection images, which were generated from a random model. By using the steepest descent method, the estimated center and angular parameters were locally to minimize the PRs of the common lines. The 3D density map was determined using the refined center and angular parameters. Cross-correlation between the particle image and its corresponding image generated from the map computed the center parameter. Then, the new center parameter was obtained to estimate the angular parameter through another global search of the icosahedral asymmetric unit. We iterated the global search, local refinement of the center and angular parameters, and 3D reconstruction, meanwhile, structural resolution was increased step by step using the gold-standard procedure [57].

### 2.3. Atomic Model Building and Refinement

With the aid of bulky residues and the structural models predicted using PSIPRED secondary structure prediction [58], the models of nine MCP monomers and nine CP monomers in the asymmetric unit of the icosahedron were manually built based on our cryo-EM density map using COOT software [59]. The models were refined using real-space refinement implemented in Phenix [60]. The refinement and validation statistics are shown in Table S1. All figures were prepared with Chimera [61]. In all electrostatic potential surfaces, the blue represents 10 kcal/(mol e^−^) positive potential, whereas the red represents −10 kcal/(mol e^−^) negative potential.

## 3. Results

### 3.1. Overall Structure of the GP4 Head

**Figure 1:**
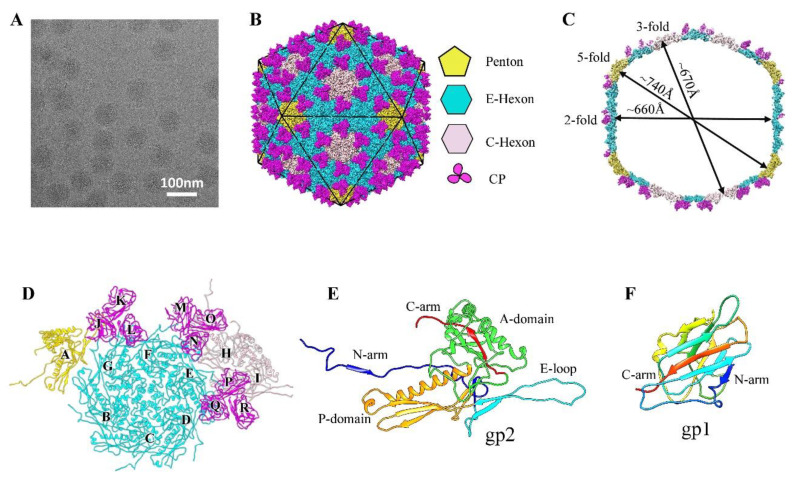
Overall structure of the GP4 head. (**A**) A representative cryo-EM image of the frozen-hydrated GP4. (**B**) Overall density map of the mature GP4 head. The densities of pentons, E-hexons, C-hexons, and cement protein trimers are colored gold, cyan, pink, and magenta, respectively. (**C**) Central cross section of the density map viewed along an icosahedral two-fold axis. The diameters of the outermost surface along the icosahedral five-, three-, and two-fold axes are labeled. (**D**) The ribbon models of the 9 MCP monomers (labeled A–I) and 9 CP monomers (labeled J–R) in the asymmetric unit of the icosahedron. The view and the color schedule are the same as in (**B**). (**E**) Secondary structure of the MCP gp2 shown in four domains: N-arm in blue, E-loop in cyan, P-domain in orange, and A-domain in green and red. The C-terminal of gp2 is colored red. (**F**) Secondary structure of the CP gp1 rainbow-colored from the N-terminus in blue to the C-terminus in red.

The mature GP4 phage was purified from *Ralstonia* cells for cryo-EM imaging. We collected 3921 micrographs of GP4 using an FEI Tecnai Arctica 200-kV electron microscope equipped with a Falcon II camera (Figure 1A). Data collection and reconstruction statistics are shown in Table S1. We selected a total of 40,792 particle images in total and performed three-dimensional (3D) reconstruction with icosahedral symmetry imposed [53]. Finally, we obtained a 3.7 Å resolution density map of the GP4 head that allowed us to build the full atomic models of the MCPs and CPs in the asymmetric unit of the icosahedron (Figure 1B–F and Figure S1A–E). The effective resolution of our structure was estimated using the gold-standard Fourier shell correlation criterion [62]; the map applies a mask with an outside diameter of 385 Å and an inside diameter of 295 Å (Figure S1F).

The head of GP4, with a maximum diameter of approximately 740 Å along the two opposite fivefold vertices, is composed of MCP gp2 and CP gp1, 540 copies each, forming an icosahedron with T = 9 (Figure 1B,C). Owing to the larger T number, the diameter of the GP4 head is larger than that of a head with T = 7 (~650Å), such as HK97, T7 [17], and BPP-1 [21]. The 540 copies of the MCPs were arranged in 12 pentons and 80 hexons forming a complete icosahedral shell without any noticeable gap. The 12 pentons occupy the 12 vertices of the icosahedron. Of the 80 hexons, 20, termed C-hexons, were located at the center of the 20 faces of the icosahedron, and the remaining 60 hexons, termed E-hexons, were located on the 30 edges of the icosahedron (Figure 1B). The 540 copies of the CPs were arranged into 180 trimers attached to the quasi-threefold sites to further stabilize the shell. Therefore, the asymmetric unit of the icosahedron contains 9 monomers of gp2, which include one E-hexon, one-third C-hexon, and one-fifth penton and the 9 monomers of gp1 (Figure 1D).

### 3.2. Structure of the Major Capsid Protein

We traced 366 (Ser2 to Asn367) of the 369 amino acid residues of the MCP gp2 in our cryo-EM density map (Figure 1E and Figure S1D). Consistently, the overall structure of gp2 has a striking resemblance to that of the MCP gp5 in HK97 [12] and the floor domain of MCP VP5 (residues 2–396 and 1059–1116) in HSV-1 [7], although only sharing a 10% sequence identity (Figure S2). According to the gp5 domain nomenclature in HK97, each gp2 monomer can be divided into four domains (Figure 1E): an especially extended N-arm domain (residues 2–47), a long β-hairpin E-loop domain (residues 48–91), a P-domain (residues 92–140 and 307–342) containing two α-helices and a three-stranded β-sheet, and a highly complex A-domain (residues 141–307 and 343–367) including the long C-arm (residues 348–367). The MCP structure of GP4 is similar to that of most known tailed phages (Figure S3), such as SF6 [18], T4 [33], phiRSA1 [44], P74-26 [27], and BPP-1 [21].

**Figure 2:**
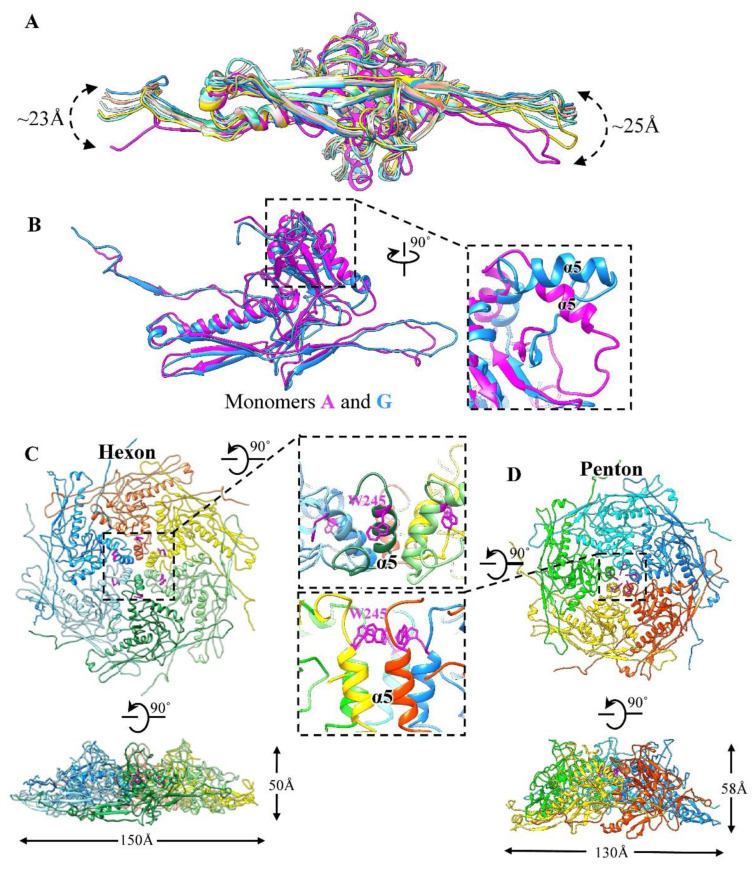
Structure of MCPs. (**A**) Superimposition of the nine MCPs with different colors showing global conformational changes. (**B**) Superimposition of monomers A and G with the same color schedule but a different view to (**A**). The inset is a zoom-in view of the conformational changes of α5 (residues 230–278) in the A-domain. (**C**,**D**) The hexon and penton (six and five subunits in different colors) are shown along the top and the side views, respectively. The inset is a zoom-in view of the local region of the hexon/penton A-domains where the side chains of residue W245 (magenta) are shown as stick models.

The superposition of the nine monomers (named A-I) of gp2 in the asymmetric unit of the head shows significant conformational changes in the N-arms and E-loops resulting in large-scale swinging (Figure 2A). The helix α5 (residues 245–255) of monomer A (from penton) adopts a drastically different conformation to the other eight monomers (Figure 2B), which results in a greater curvature in the central conformations of the pentons than in the hexons (Figure 2C,D). Extensive and complex interactions were formed between inter-capsomeres, as seen in other phages, such as SF6 [18], Mic1 [25], and HK97, via contact between the A-domains to form the inner circumference at the center, as well as the peripheral interactions between adjacent E-loops and P-domains, as seen in the hexon, for example (Figure S4A). Meanwhile, the N-terminal loop (residues 14–19) reaches the neighboring monomer in the same capsomere to form a three-stranded β-sheet (Figure S4A). As reported previously, lambda [63] and YSD1 [64] also form a stabilizing β-sheet in the capsomeres. Altogether, the structural differences in MCPs enable gp2 monomers to form different conformational capsomeres, including pentons, E-hexons, and C-hexons, resulting in the formation of an icosahedral capsid. Notably, the E-hexons and C-hexons exhibit mildly different conformations, where the E-hexon has a slight bent structure and the C-hexon is apparently flat, mainly mediated by the curvature to build the capsid, as seen in A-1(L) [24] and KHP30 [65] owing to a highly similar arrangement of MCPs with a T number of 9 (Figure S4B).

Previous studies indicate that the MCPs arrange to form different capsomers, pentons, and hexons via conformation changes in the A-domains located at the capsomer centers, such as the α-helix-to-β strand structural transition in SF6 [18], β-to-loop transition in phage Mic1 [25], and the α-helix-to-loop transition in phi29 [15]. It is interesting to note that the curvature of the GP4 penton, with an external ~130 Å diameter and a ~58 Å height, was obviously greater than that of the hexon, which had an external ~150 Å diameter and a ~50 Å height (Figure 2C,D). The α5 helices in the A-domains are perpendicular to the capsid surface at the center of the penton instead of the incline in the hexon (Figure 2C,D). Accompanying the movements of the α5 helices, the five bulky sidechains (Trp245) in the penton point to the central pore and form a hydrophobic core. In contrast, the sidechain Trp245 in the hexon flips to the opposite direction and inserts into a hydrophobic pocket formed by Leu237, Leu246, Pro261, Ile262, and Leu267 in the same MCP monomer (Figure S4A). In addition, the penton shows a regular distribution of electrostatic potential along the junction between the two monomers on the inner surfaces but the hexon shows a discrete distribution of charges (Figure S5).

### 3.3. Structure of Cement Proteins

**Figure 3:**
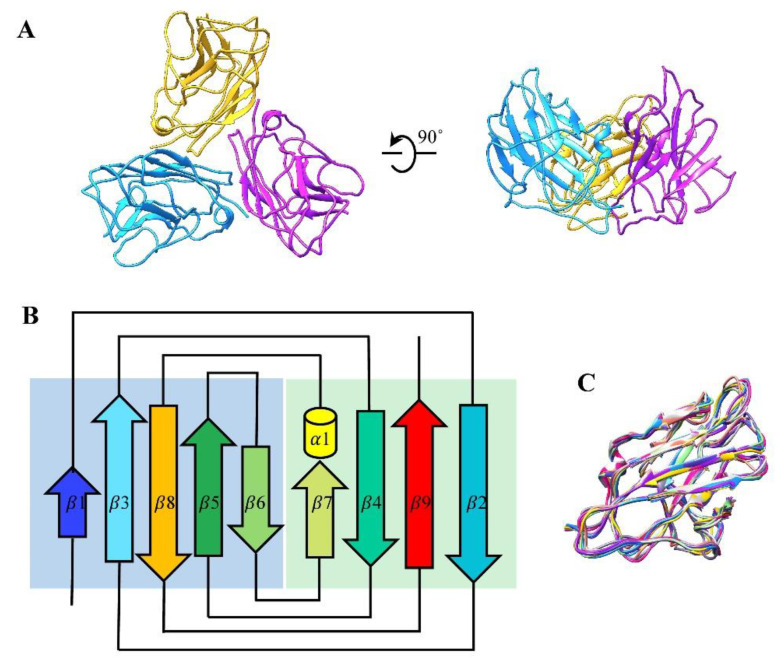
Structure of CPs. (**A**) Ribbon model of the CP trimer gp1 shown in top and side views. (**B**) Topological diagram of gp1. The color scheme is the same as in Figure 1F. (**C**) Superimposition of the nine CP monomers colored differently, with negligible differences.

We traced 155 (Ala2 to Ser156) of the 157 amino acid residues of CP gp1 in our cryo-EM density map (Figure 1F and Figure S1E). We found that 540 monomers of gp1 form 180 trimers that stick on the outer surface of the capsid along the quasi-three-fold axes of the icosahedron (Figure 1B and Figure 3A). Each gp1 monomer contains a short α-helix and two β-sheets; the two β-sheets consist of four anti-parallel strands and five parallel strands (Figure 3B). The structure of the nine CP monomers (named J-R) in the asymmetric unit are basically identical (Figure 1D and Figure 3C), which is also true of the nine CPs of KHP30 [65]. Comparisons of the CP structure of GP4 and other phages, such as BPP-1 [21], ε15 [19], KHP30, and Pam1 [20], indicate that the GP4 CPs adopt a similarly topological structure to multiple immunoglobulin (Ig)-like folding motifs (Figure S6A). Although the Ig-like topological structures are strikingly different from that of other typical cement proteins, such as Lambda gpD, which has a tulip fold [26]; phage L Dec protein, which has an OB-fold [66]; and T4 Soc, which has a β-Tadpole fold [67], all of these evolved from a common ancestor [10].

In some phages, mechanical weak points exist at the three-fold axes and/or the quasi-three-fold axes; hence, the unique interaction or augmentation that extra cement proteins apparently play in reinforcing the capsid [27,68]. HK97 utilizes a covalent bond to link the trimeric capsomeres in the threefold and the quasi-threefold axes [12]. A-1(L) utilizes additional domains, L-loops from MCPs situated at the inner surface of the capsid around the quasi-three-fold axes, to form a three-layered interaction interface, including L-loops, P-loops, and E-loops [24]. However, phages such as lambda, GP4, KHP30, and Pam1, utilize the trimeric CPs to stabilize the capsid at the three-fold axes and/or the quasi-three-fold axes, but there are significant differences in the interactional modes between CPs and MCPs (Figure S6B–E). In the case of Lambda [63], gpD possesses an elongated N-terminal interacting with the E-loop of the MCPs and the N-terminal of the neighboring MCPs, forming a stabilizing four-stranded β-sheet. Furthermore, all gpDs, via intricate interactions, form an icosahedral cage around the head. In KHP30 with T = 9 [65], the CP contains an elongated N-terminal and C-terminal forming an anti-parallel two-stranded β-sheet. The β-sheet is located at the bottom of the adjacent monomer in the CP trimer and interacts with the N-arm and E-loop of the MCPs around the quasi-three-fold axes. In Pam1 with T = 7 [20], the CP has an elongated N-terminal loop, which extends to the bottom of the adjacent monomer in the CP trimer. A significant perpendicular interaction exists between the N-terminal loop of the CPs and the E-loop of the MCPs around the three-fold axes and/or the quasi-three-fold axes. Compared with Pam1, in Lambda and KHP30, the N- and C-termini of the GP4 CPs form stable interactions with the trimeric body without obvious extension.

Three acid residues (Asp23) in the trimeric CP point to the axial center of the trimer and are located on the lower surface of the trimer, separated by a distance of approximately 3Å (Figure S7). Simultaneously, a dense sphere of possible metallic cations is surrounded by the three sidechains (Asp23). Such a metal cation interacts with three Asp23 residues to form a binding site, probably generated by SM buffer (10 mM Tris–HCl, pH 7.5, 100 mM NaCl, and 10 mM MgSO4) in the neutral environment, reinforcing the stability of trimeric CPs (Figure S7). Similar structures were observed in HK97 [69]. Both the centers of hexamers and pentamers in HK97 contain a dense sphere corresponding to a sulphate ion and a chloride ion, respectively. Both ions interact with sidechains (Arg294) in HK97 and the anion exists in the crystallization buffer.

### 3.4. Interactions among Capsid Proteins

**Figure 4:**
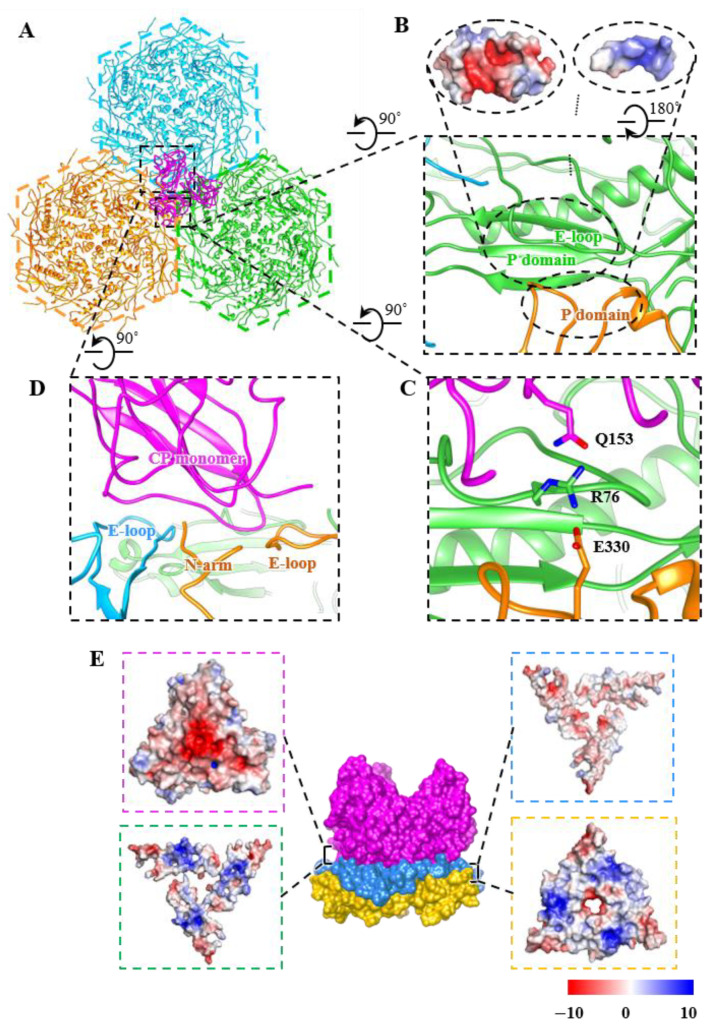
Interactions between the trimer and hexon capsomeres around the quasi-three-fold axis. (**A**) Top view of the interactions along a quasi-three-fold axis. The three hexon capsomeres and the CP trimer are colored orange, deep sky blue, lime green, and magenta, respectively. (**B**) Zoom-in view of the inter-capsomeric interactions mediated by two adjacent capsomeres in the absence of the CPs. The insets show the interfaces of the interaction colored according to electrostatic potential. (**C**) Salt-bridge interactions between E330 sidechains from one capsomere and R76 from the adjacent capsomere and H-bound interaction between sidechains R76 and Q153 from CP trimers. (**D**) A single CP monomer interacting with three MCP monomers derived from two adjacent capsomeres. (**E**) Three-layered sandwich structure along a quasi-three-fold axis. The outer layer (magenta), middle layer (dodger blue), and inner layer (gold) are formed by the CP trimer, partial N-arms and E-loops of MCP, and partial P-domains of MCPs, respectively. The four inset panels show the electrostatic potential of each interface, in detail, magenta frame: the lower surface of the outer layer; green frame: the upper surface of the middle layer; blue frame: the lower surface of the middle layer; yellow frame: the upper surface of the inner layer.

Around the quasi-three-fold axis, the GP4 forms extensive non-covalent interactions along the capsomer–capsomer and the capsomer–trimer interfaces (Figure 4A). In the absence of the CPs, the contact mode of the three capsomeres is repeated in each pair of adjacent capsomeres along the three-fold axis. The predominantly positively charged P-domain (residues 113–130 and 325–330) of the MCP derived from one capsomere is inserted into a groove formed by the mainly negatively charged adjacent P-domain and E-loop from adjacent capsomers, resulting in strong complementary electrostatic interactions along the groove (Figure 4B). Simultaneously, the sidechain Arg76 in the E-loop from one capsomere interacts with Glu330 in the P-domain of the adjacent capsomere to form the salt bridge, further increasing the capsid stability (Figure 4C). Within the context of the CPs, the bottom of each CP monomer interacts extensively with three MCP monomers (Figure 4D). Especially the sidechain Gln153 of the C arm in CPs is also near the Arg76 of the E-loop in the MCP, thereby generating the H-bond to reinforce the capsid assembly around the quasi-three-fold axes (Figure 4A,C).

In addition, along each quasi-three-fold axis, a three-layered sandwich interface structure is formed by complex electrostatic interactions to maintain the stability of the quasi-three-fold axis (Figure 4E). The outer layer is formed by the bottom of the CP trimer. The middle layer is constituted by the E-loop (residues 54–94) and N-arm (residues 2–15) from different capsomeres, acting as a pedestal to support the CPs. The inner layer consists of P-domains (residues 95–137 and 316–339) from the surrounding capsomeres. These three-layered sandwich structures have also been observed in A-1(L) and P23-45 [70]. Meanwhile, the capsomer–capsomer and the capsomer–trimer structural interactions in GP4 can partially explain the stable head structure of GP4, which contributes to its survival in high-temperature environments ranging from 40 °C to 70 °C [39].

## 4. Discussion

Here, we resolved the head structure of *Ralstonia solanacearum* GP4 at 3.7 Å resolution using cryo-EM. The GP4 head, with a T number of 9, is composed of MCP gp2 and CP gp1, which exhibit a canonical HK97-like fold and an Ig-like fold, respectively. The 540 monomers of gp2 are arranged in three types of capsomeres, penton, E-hexon, and C-hexon, as well as exhibiting different conformations. The 540 monomers of gp1 form 180 trimers sticking on the outside surface at the quasi-three-fold axes of the icosahedron. The three-layered electrostatic complementary potential formed along the quasi-threefold axis by the CP trimer and its adjacent three capsomeres reinforces the capsid assembly. Additionally, given the structural similarities observed among all known tailed phages, such as Mic1 [25], SPP1 [71], T4 [72], and T5 [38], which exhibit similar phage head assembly patterns, we hypothesized the GP4 shell assembly (Figure S8). In the context of the 12-fold symmetric portal, scaffold proteins, functioning as chaperones, recruit the penton, E-hexon, and C-hexon and capsomeres form a close icosahedral capsid without an obvious gap. Then, each of the three hexamers are glued via a trimer CP by electrostatic complementary potential at the quasi-threefold axis. GP4 exhibits a stable and rigid shell in the end.

In summary, we show for the first time a solved T = 9 icosahedral head for short-tailed *Ralstonia solanacearum* phages at a high-resolution structure, compared with other *Ralstonia solanacearum* phages with different T numbers (as already mentioned above), although all of which are a HK97-like fold. The GP4 head is another example of the unique diversity of the capsids, providing important structural insights into the study of *Caudovirales* capsids. The atomic structure of the GP4 head facilitates further investigations into the structure–function relationships between GP4 and its host.

## Data Availability

The electron density maps and atomic coordinates have been deposited in the EM Data Bank and Protein Data Bank under accession codes EMD-34539 and PDB ID: 8H89, respectively.

## Supplementary Materials

The following supporting information can be downloaded at: https://www.mdpi.com/article/10.3390/v14112431/s1, Figure S1: Reconstruction scheme; Figures S2 and S3: Comparisons of the difference MCPs; Figure S4: The structure of the hexon; Figure S5: The electrostatic potential; Figure S6: Comparisons of the difference CPs; Figure S7: The spherical density; Figure S8: Schematic diagram of the capsid assembly; Table S1: Reconstruction and refinement statistics.
