# Supplementary material for: A Capsid Structure of Ralstonia solanacearum podoviridae GP4 with a Triangulation Number T = 9
Source: Viruses. 2022 Nov 1;14(11):2431. doi: 10.3390/v14112431 (PMC9698820; doi:10.3390/v14112431)
Supplement: Supplementary file 1 [file viruses-14-02431-s001.zip › viruses-1972526-supplementary.pdf]

Supplemental Information

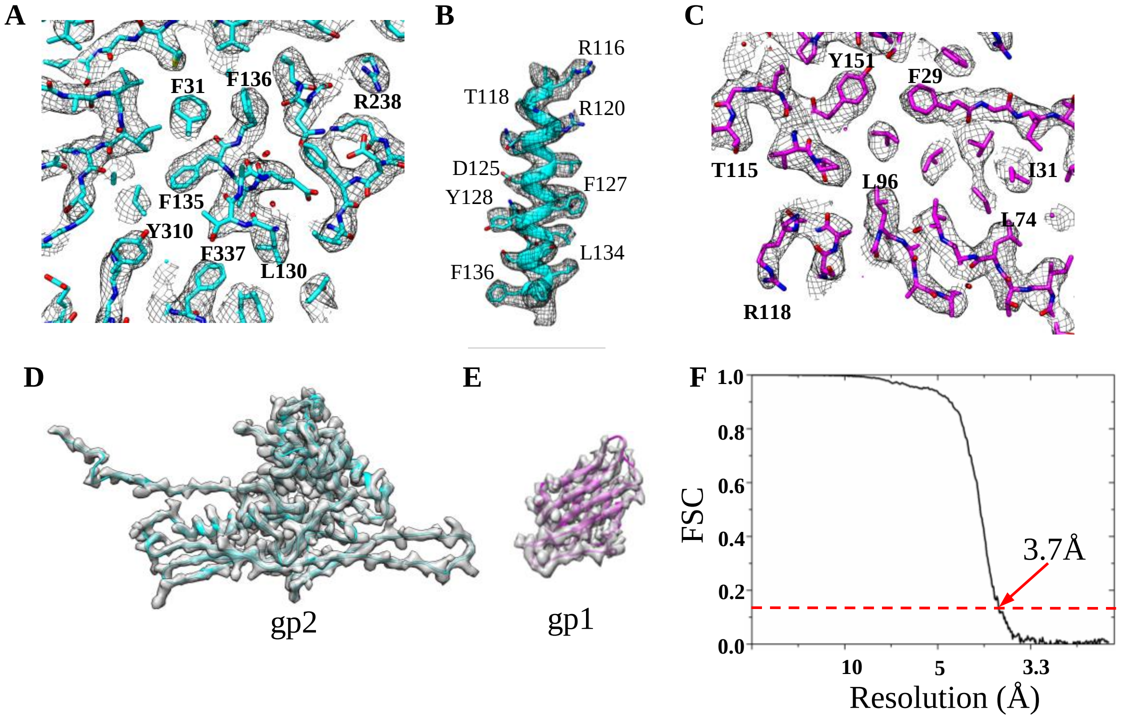

Figure S1. Quality of the cryo-EM density maps and atomic models. (A-C) Superposition of our atomic model on the densities (mesh) extracted from major capsid protein gp2 (cyan) and cement protein gp1 (magenta). (D-E) The density maps (grey) of gp2 and gp1 superimposed on their atomic models (ribbons), respectively. (F) The Fourier shell correlation (FSC) curve of the GP4 head map.

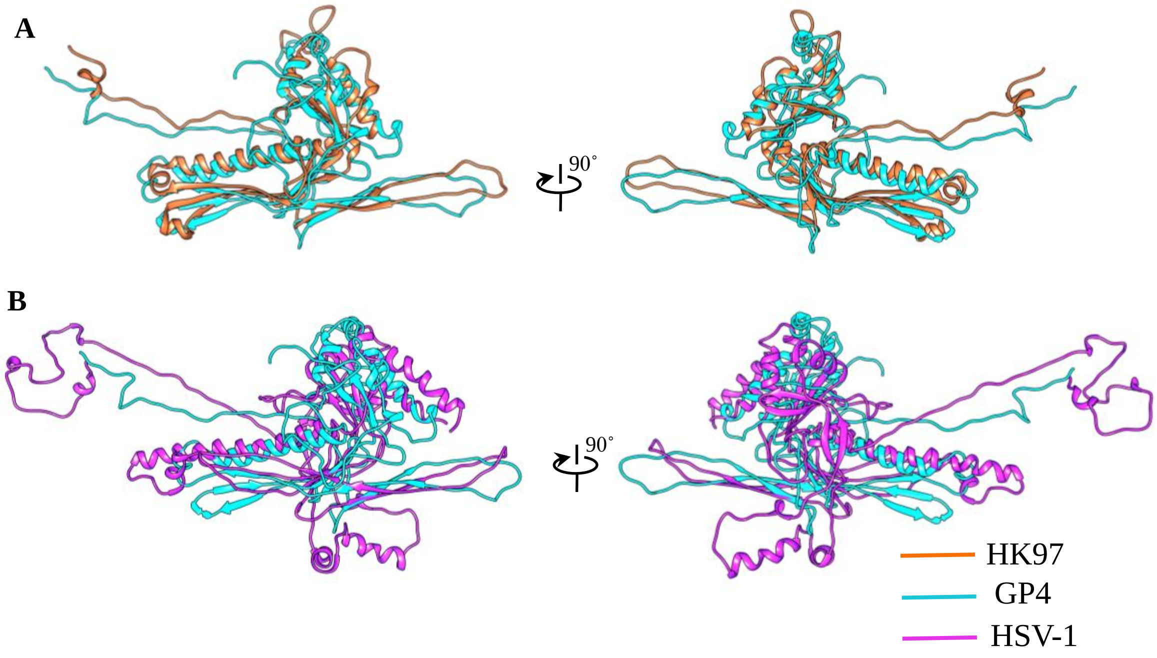

Figure S2. Comparisons of the secondary structure of the GP4 MCP monomer with HK97 (PDB ID: 1OHG) MCP monomer (A) and the floor domain of HSV-1 (PDB ID: 6CGR) MCP monomer (B). The RMSD by using Dali server of their MCP backbones between the GP4 and HK97, the floor domain of HSV-1 is 3.9 Å, 4.3 Å, respectively.

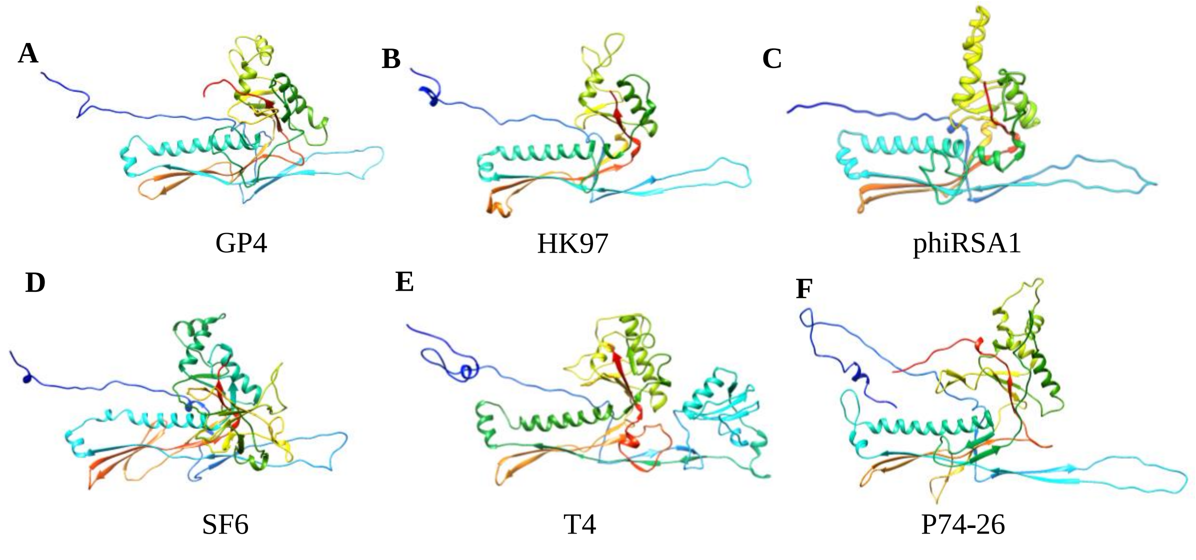

Figure S3. Comparison of MCPs of GP4 and other phages. (A) GP4. (B) HK97 (PDB ID: 1OHG). (C) phiRSA1 (PDB ID: 7OZ4). (D) SF6 (PDB ID: 5L35). (E) T4 (PDB ID: 5VF3). (F) P74-26 (PDB ID: 6O3H). Secondary structures are rainbow-colored from the N-terminus in blue to the C-terminus in red. The RMSD of their MCP backbones between the GP4 and HK97, phiRSA1, SF6, T4, P74-26 is 3.9 Å, 4.6 Å, 4.7 Å, 5.6 Å, 3.6 Å, respectively.

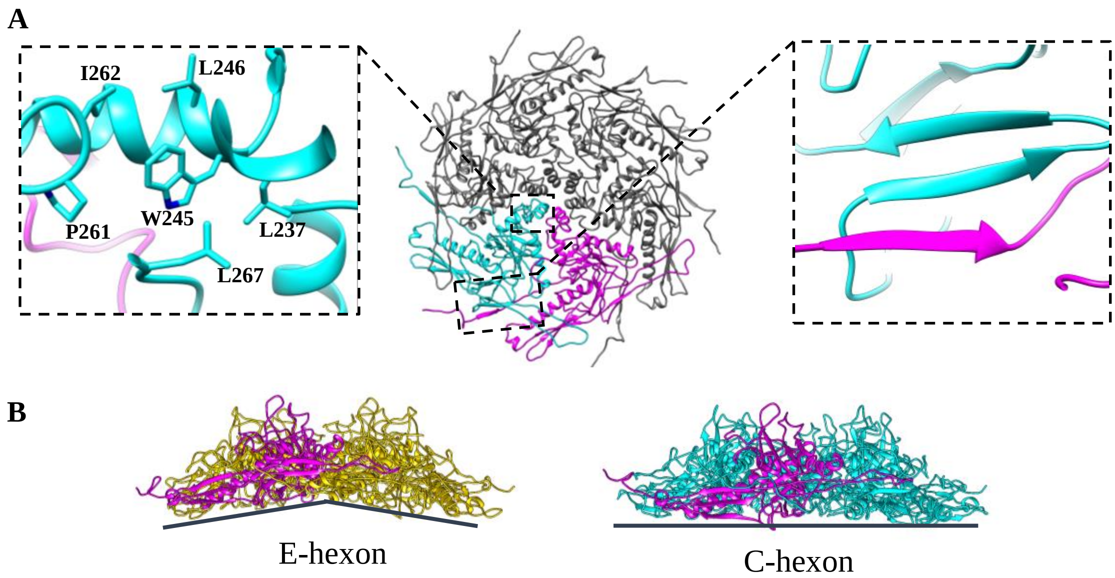

Figure S4. Structure of the hexon. (A) The interactions among MCP monomers in a hexon. The left inset shows a zoom-in view of hydrophobic group, and the right inset shows a zoom-in view of an increased β sheet. (B) Structural comparison between E-hexons and C-hexons viewed laterally.

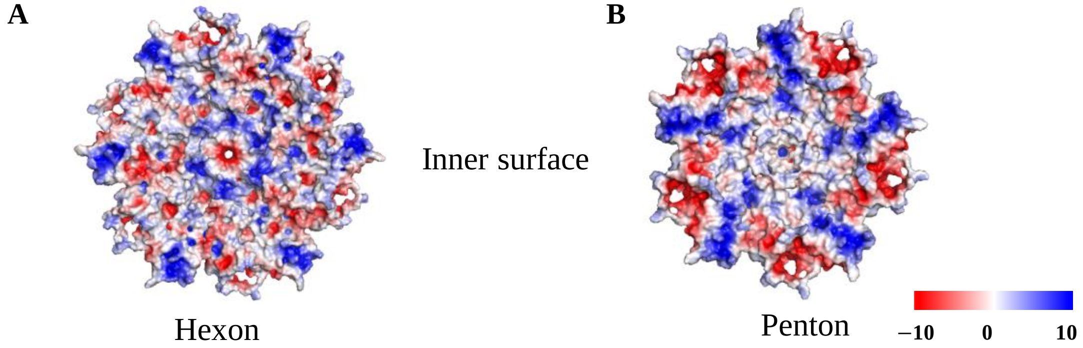

Figure S5. The capsomeric surfaces colored according to electrostatic potential. (A) The hexon shares one discrete interaction interface. (B) The penton shows a regular interaction interface. The electrostatic potential scale is shown in the color bar.

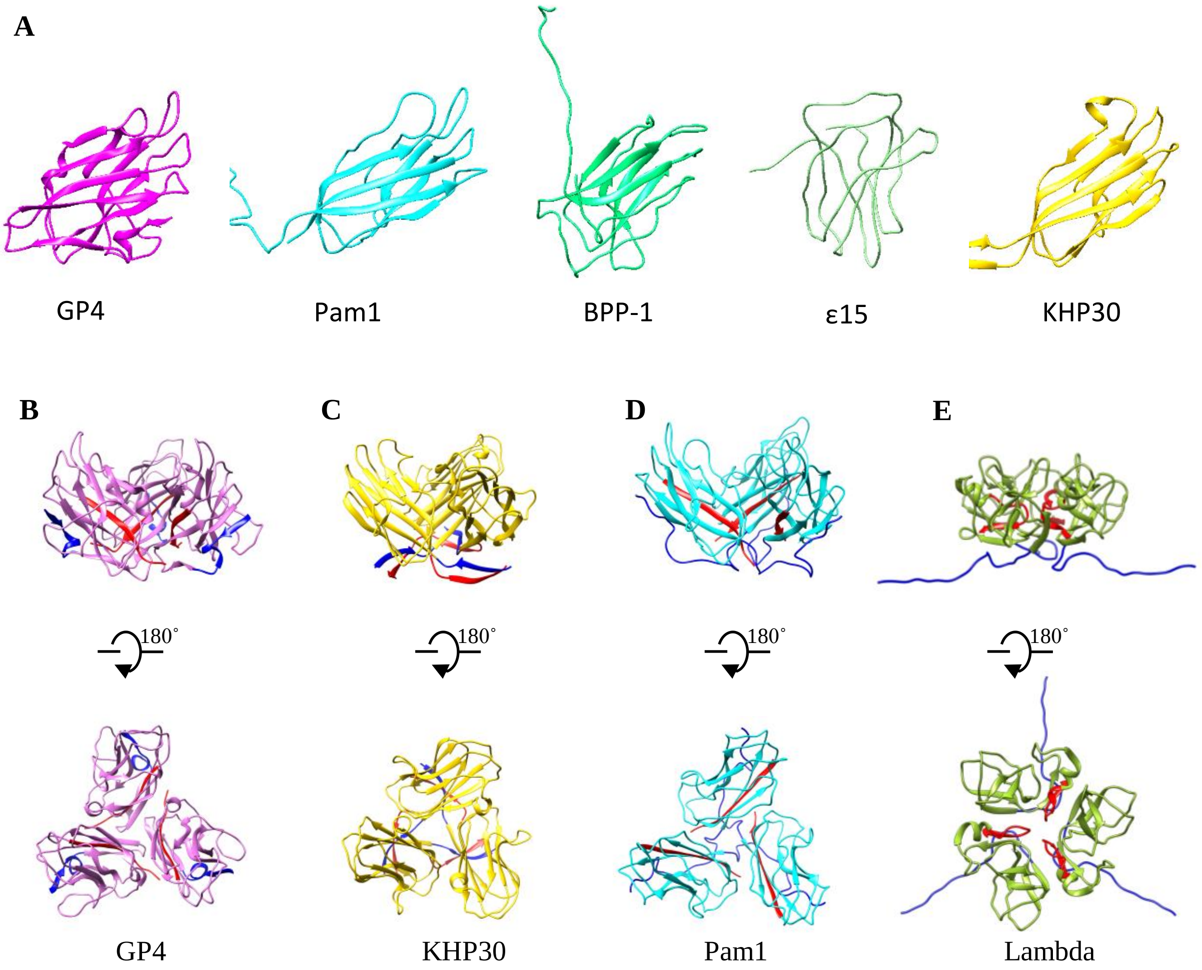

Figure S6. Structural comparisons of CPs in GP4 and other phages. (A) Ribbon model of GP4 CP compared with Pam1 (PDB ID: 7EEL), BPP-1 (PDB ID: 3J4U), ε15 (PDB ID: 3J40), KHP30 (PDB ID: 7DN2). The RMSD of their CP backbones between the GP4 and Pam1, BPP-1, KHP30 is 3.0 Å, 3.0 Å, 2.1 Å, respectively, with the exception of ε15. (B) The structure of monomer is rainbow-colored from the N-terminus in blue to the C-terminus in red (left). The CP trimer is shown in side view (up) and top view (below). The trimer is colored in hot pink except for N termini in blue and C termini in red. (C-E) Ribbon models of phages KHP30, Pam1 and Lambda. The trimeric Dec of KHP30, Pam1 and Lambda are colored yellow, cyan and olive drab, respectively, and the other color schedules and directions of view are same as in panel B.

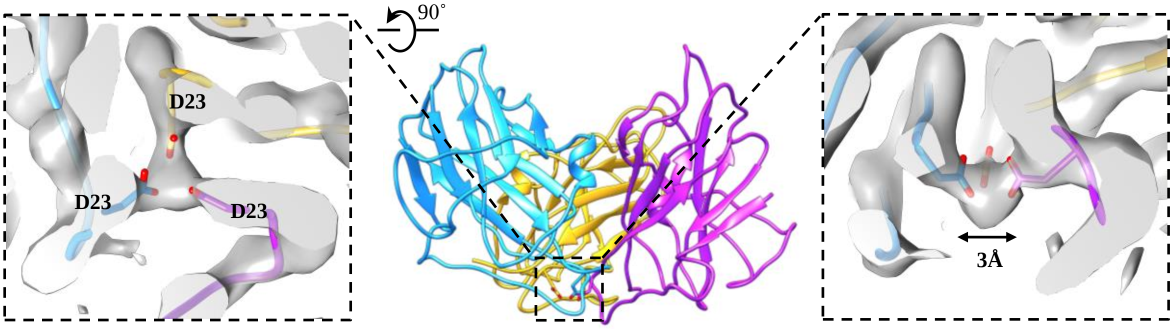

Figure S7. Spherical density at the center of trimeric CP.

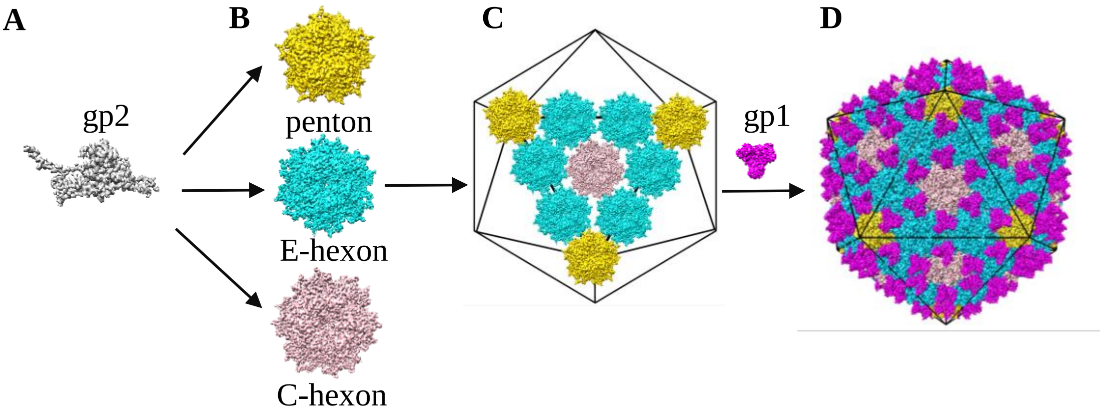

Figure S8. A schematic diagram of the GP4 capsid assembly. (A) The MCP gp2 (gray). (B) The gp2 monomers are organized into three types of capsomeres: pentons (gold), E-hexons (cyan), and C-hexons (pink). (C) Several capsomeres are shown on a single face of an icosahedron. (D) Recruitment of the CP gp1 (magenta) around the quasi-three-fold axial sites further enhances the stability of the capsids.

Table S1. Cryo-EM parameters, data collection, refinement statistics.

Data collection		
Elcetron microscopy	FEI 200 kV Technai Arctia, Falcon II camera	
Pixel size(Å)	1.27	
Defocus range	0.1 to 3.8µm	
Voltage(KeV)	200 kV	
Total movie-mode micrographs	3921	
**Icosahedral reconstruction**		
Total particles	40792	
Resolution	3.7Å	
B-factors	270	
EMDB ID	EMD-34539	
**Atomic models refinement/statistics(phenix.real_space_refine)**		
Protein	gp1	gp2
PDB ID	8H89	
Model Resolution in Refinement	3.7Å	
Total Residues	1395	3306
CC_mask (model to map fit)	0.8195	0.7705
Ramachandran most favorable (%)	93.25%	95.65%
Ramachandran additional allowed (%)	6.46%	4.32%
Ramachandran disallowed (%)	0.29%	0.03%
